# Impact of Intraoperative Lidocaine During Oncologic Lung Resection on Long-Term Outcomes in Primary Lung Cancer: A Post Hoc Analysis of a Randomized Controlled Trial

**DOI:** 10.3390/cancers17172923

**Published:** 2025-09-06

**Authors:** Elena de la Fuente, Francisco de la Gala, Javier Hortal, Carlos Simón, Almudena Reyes, Lisa Rancan, Alberto Calvo, Angela Puig, Elena Vara, José María Bellón, Patricia Piñeiro, Ignacio Garutti

**Affiliations:** 1 Department of Anesthesiology, Gregorio Marañón University Hospital, 28007 Madrid, Spainignacio.garutti@salud.madrid.org (I.G.); 2 Biomedical Research Institute, Gregorio Marañón University Hospital, 28007 Madrid, Spain; 3 Research into Medical and Surgical Science Program, Faculty of Medicine, Complutense University of Madrid, 28040 Madrid, Spain; 4 Department of Thoracic Surgery, Gregorio Marañón University Hospital, 28007 Madrid, Spain; 5 Department of Biochemistry and Molecular Biology, Faculty of Medicine, Complutense University, 28040 Madrid, Spain; 6 Unit of Methodology and Biostatistics, Gregorio Marañón University Hospital, 28007 Madrid, Spain; 7 Department of Pharmacology and Toxicology, Faculty of Medicine, Complutense University, 28040 Madrid, Spain

**Keywords:** lidocaine, thoracic anesthesia, paravertebral, inflammation, lung cancer, overall survival, disease-free survival

## Abstract

**Background/Objectives**: Lidocaine has demonstrated immunomodulatory properties and promising antitumor effects in experimental models, but its impact on long-term outcomes following oncologic surgery remains unclear. This study aimed to compare the impact of intraoperative lidocaine versus remifentanil on long-term cancer outcomes after primary lung cancer surgery. **Methods**: This is a post hoc analysis of a randomized controlled trial (NCT03905837, EudraCT 2016-004271-52). From 154 patients who underwent elective lung resection via video-assisted thoracoscopic surgery (VATS) between January 2019 and June 2021 and were randomized to receive intraoperative lidocaine (intravenous or paravertebral) or remifentanil, we analyzed data from patients with confirmed primary lung cancer in the surgery specimen. Overall survival (OS) and disease-free survival (DFS) were assessed through May 2025. Survival outcomes were analyzed using Kaplan–Meier curves and log-rank tests. A multivariate Cox proportional hazards model was used to adjust for potential confounders. **Results**: Among the 97 patients with primary lung cancer finally included in the analysis, those in the lidocaine group exhibited improved OS compared with those who received intravenous remifentanil (log-rank *p* = 0.022). This association remained significant in the multivariate Cox regression analysis (HR 2.59, 95% CI 1.13–5.96, *p* = 0.025). No significant differences were observed in DFS overall (log-rank *p* = 0.283) or in DFS limited to recurrences of cancers present at the time of surgery, either the resected primary tumor or a prior malignancy (log-rank *p* = 0.080). **Conclusions**: In this post hoc analysis, lidocaine administration during oncologic lung resection was associated with improved OS in primary lung cancer patients. No differences in DFS were observed between groups; however, a non-significant trend toward improved DFS in lidocaine patients was noted when focusing on recurrences of cancers present at the time of surgery. Further investigation in larger prospective studies is warranted.

## Simple Summary

Lidocaine has demonstrated immunomodulatory properties and, additionally, has shown promising antitumor effects in experimental models. However, its impact on long-term outcomes following oncologic surgery remains unclear. This is a post hoc analysis of a randomized controlled trial. Of 154 patients undergoing lung resection surgery randomized to receive either intraoperative lidocaine (intravenous or paravertebral) or remifentanil, data from 97 patients with confirmed primary lung cancer were analyzed to compare overall survival and disease-free survival. In our study, intraoperative lidocaine administration was associated with improved overall survival compared with remifentanil, without significant differences in disease-free survival. However, when considering only recurrences of pre-existing cancers at the time of surgery (either the resected primary tumor or previous malignancies), a non-significant trend toward improved disease-free survival was observed with lidocaine. Further investigation in larger prospective studies is warranted.

## 1. Introduction

Lung cancer is the leading cause of cancer-related mortality worldwide, remaining highly prevalent in both developed and developing countries. Surgical resection is the preferred treatment for early-stage non-small cell lung cancer (NSCLC) and plays a crucial role in the multidisciplinary management of pulmonary oncologic disease alongside chemotherapy, radiotherapy, and immunotherapy [1].

However, the perioperative period represents a window of vulnerability, as tumor removal may promote local and systemic dissemination of cancer cells due to surgical manipulation and vascular disruption. Additionally, the surgical stress response (SSR), while essential for tissue repair and organ function, activates neural, inflammatory, and proangiogenic pathways that may promote tumor cell seeding and proliferation [2,3]. SSR may also shift the immune profile toward a T helper cell type 2 (Th2)-dominant, antibody-mediated response, potentially weakening the Th1-mediated cytotoxic activity required to eliminate residual cancer cells [4]. Thoracic surgery is known to provoke a more pronounced inflammatory response than other types of surgery [5].

Local anesthetics (LAs) have demonstrated immunomodulatory effects [6] and antitumoral properties in experimental models [7]. Despite promising preclinical findings, clinical evidence—mainly from retrospective observational studies—remains inconclusive regarding the impact of intraoperative LA administration on long-term cancer outcomes. Two recent large randomized controlled trials failed to demonstrate improved oncologic outcomes with combined general anesthesia plus regional analgesia with LAs compared to control. The first trial, conducted in breast cancer patients, evaluated general anesthesia with a paravertebral block (PV) [8], while the second study, in lung cancer patients, assessed general anesthesia with an epidural block [9]. These discrepancies between laboratory findings and clinical trial results may stem from the inability of experimental models to fully replicate the complexity of cancer biology, including the critical role of the microenvironment and immunology [10]. Therefore, further research is needed to identify the optimal LA agent, administration route, and specific tumor types that may benefit from these potential antitumoral effects [11].

This post hoc analysis of a randomized controlled trial (RCT) aimed to compare the impact of intraoperative lidocaine administration—via either the intravenous (IV) or paravertebral (PV) route—versus remifentanil on overall survival (OS) and disease-free survival (DFS) in primary lung cancer patients who underwent elective lung resection surgery (LRS) via video-assisted thoracoscopy (VATS).

## 2. Materials and Methods

This is a post hoc analysis of a randomized clinical trial (RCT) (EudraCT 2016-004271-52, NCT03905837) that was approved by the local Clinical Investigation Ethics Committee (10/2017) in Madrid, Spain (Chairperson Dr Díaz Otero) in May 2017 [12,13]. Written informed consent was obtained from all subjects. The original trial randomized adult patients undergoing VATS-LRS at Gregorio Marañón University Hospital, between January 2019 and June 2021, to receive intraoperative lidocaine (IV or PV) versus remifentanil and assessed first-month postoperative complications and pulmonary and systemic inflammation. For this post hoc analysis, only patients with anatomopathologically confirmed primary lung cancer in the surgical specimen were included, and overall survival (OS) and disease-free survival (DFS) were assessed through May 2025.

The exclusion criteria considered were as follows: preoperative forced expiratory volume at one second or forced vital capacity < 50%, history of liver disease, pregnancy or lactation, known hypersensitivity to amide-type LAs, chronic corticosteroid treatment or immunosuppressant use in the prior 3 months, blood product transfusion within the previous 10 days, and a positive SARS-CoV-2 RT-PCR test within 48 h before surgery (from June 2020).

Patients were randomly assigned (computer-generated random numbers) to one of the following groups (medications were indistinguishable clear liquids administered at fixed rates according to ideal body weight):

1. IV-LIDO: IV lidocaine (1.5 mg/kg/h) + PV saline;
2. PV-LIDO: PV lidocaine 2% (0.1 mL/kg/h) + IV saline;
3. REMI: IV: remifentanil (0.1 mcg/kg/min) + PV saline.

After induction, a double-lumen tube was used for lung isolation, and anesthesia was maintained with sevoflurane. Following patient positioning for surgery, a PV catheter was placed at the ipsilateral T5 level, and the study drugs were initiated. Protective ventilation during one-lung ventilation (OLV) included recruitment maneuvers and optimal positive end-expiratory pressure (PEEP) titration. At the end of surgery, all study perfusions were stopped, and a 0.15 mL/kg PV bolus of 0.2% ropivacaine was administered. Withdrawal criteria included the following: inability to undergo protective ventilation and relevant deterioration during study drug administration (e.g., severe hypotension refractory to vasopressors or sudden life-threatening arrhythmia).

Postoperatively, all patients were managed according to the Enhanced Recovery After Surgery (ERAS) program established at our center. Multimodal analgesia included 0.2% PV ropivacaine perfusion for the first 48 h, nonsteroidal anti-inflammatory drugs (NSAIDs) unless allergic, and morphine as rescue analgesia [14].

Data were collected from electronic hospital records or, in cases where the patient had moved to another healthcare facility, through telephone interviews. The Thoracic Surgery and Oncology departments followed up on patients according to standard protocols at our center. DFS was defined as the number of days from surgery to the first diagnostic test indicating oncologic recurrence, including relapse of the current primary lung cancer, recurrence of previous cancer, or any new primary cancer. Overall survival (OS) was defined as the time from surgery to death from any cause. Investigators responsible for data collection, processing, and analysis were blinded to group assignments.

### Statistical Analysis

Continuous variables are reported as median (interquartile range). Quantitative variables were compared using Student’s *t*-test or the Mann–Whitney U-test. Normality was assessed using the Kolmogorov–Smirnov test. Categorical variables are presented as frequencies and percentages, and groups were compared using Pearson’s chi-squared test or Fisher’s exact test. Survival outcomes were analyzed using Kaplan–Meier curves and compared with the log-rank test. Multivariate explanatory analysis was performed using Cox proportional hazards regression models to adjust for potential confounders. To minimize the risk of overadjustment, each multivariate model was limited to a maximum of four covariates. In Model A, variables were selected a priori based on clinical relevance: American Society of Anesthesiologists Physical Status Classification (ASA) > II (representing overall comorbidities), duration of surgery > 180 min (surgical factors), and lung cancer stage > I (oncologic factors). In Model B, covariates were selected based on statistically significant associations identified in univariate Cox regression (*p* < 0.1), while ensuring the absence of meaningful collinearity among variables. Statistical analyses were conducted using SPSS Statistics v28.0.1.1 (IBM Corp, Armonk, NY, USA) and Stata v19 (StataCorp., College Station, TX, USA). Statistical significance was set at *p* < 0.05.

## 3. Results

### 3.1. Study Population

**Figure 1:**
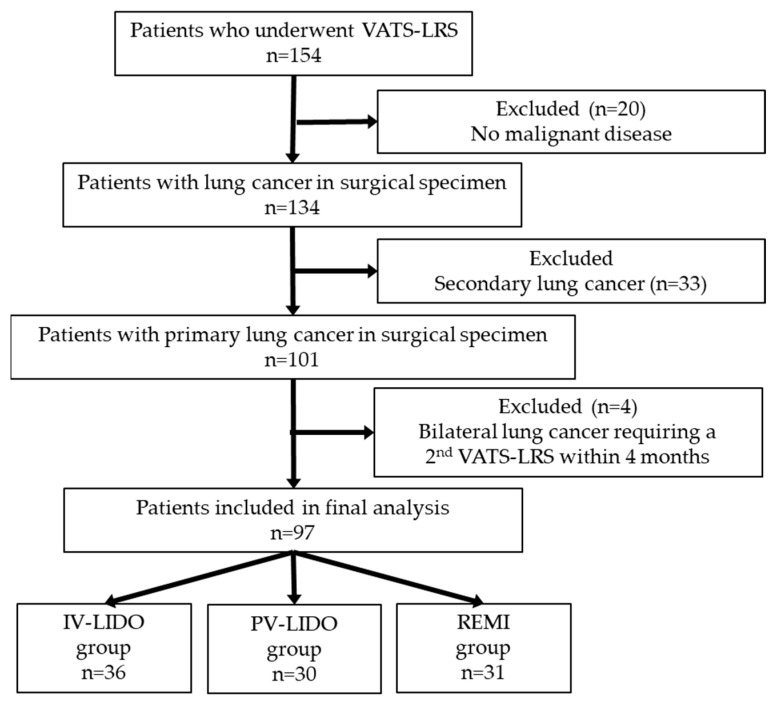
Flow chart. VATS, Video-Assisted Thoracoscopy; LRS, lung resection surgery; IV, intravenous; PV, paravertebral; LIDO, lidocaine; REMI, remifentanil.

Of 154 patients analyzed in the RCT, 97 patients had an anatomopathological diagnosis of primary lung cancer in the surgery specimen. Four patients from the lidocaine group were excluded due to bilateral lung disease that required a second VATS-LRS under OLV within 4 months (Figure 1).

The lidocaine and remifentanil groups were comparable in terms of demographics, comorbidities, surgical procedures, and oncologic characteristics. The only significant differences between the randomization groups were observed in the incidence of complications. Patients in the lidocaine group experienced fewer major complications (according to the Clavien–Dindo classification [15]), as well as fewer pulmonary and cardiac complications, compared with the remifentanil group (Table 1).

### 3.2. OS and Death Causes

**Figure 2:**
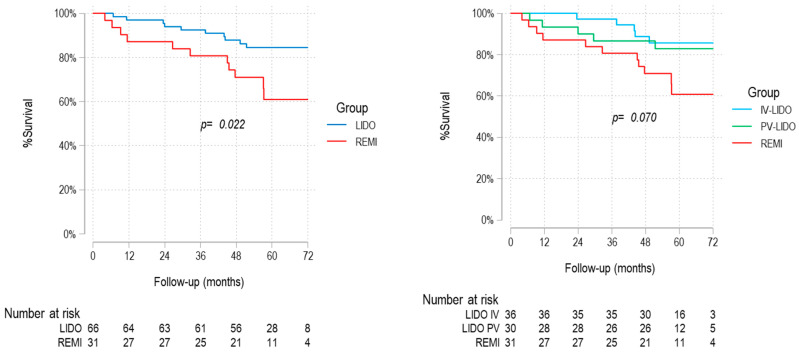
Kaplan–Meier OS curves. IV, intravenous; PV, paravertebral; LIDO, lidocaine; REMI, remifentanil.

Patients receiving intraoperative lidocaine presented improved OS compared with those who received IV remifentanil (log-rank *p* = 0.022) (Figure 2). This association remained significant in multivariate Cox regression analyses, both in a model including preselected covariates (Model A: hazard ratio (HR) 2.59; 95% confidence interval (CI) 1.13–5.96; *p* = 0.025) and in a model including variables with significant associations in univariate analyses while ensuring the absence of meaningful collinearity (Model B: HR 5.41; 95% CI 1.86–15.72; *p* = 0.002) (Table 2). Except for one pneumonia-related death in the remifentanil group, all one-year deaths were attributed to cancer progression (Table 3).

### 3.3. DFS and Recurrence Origin

**Figure 3:**
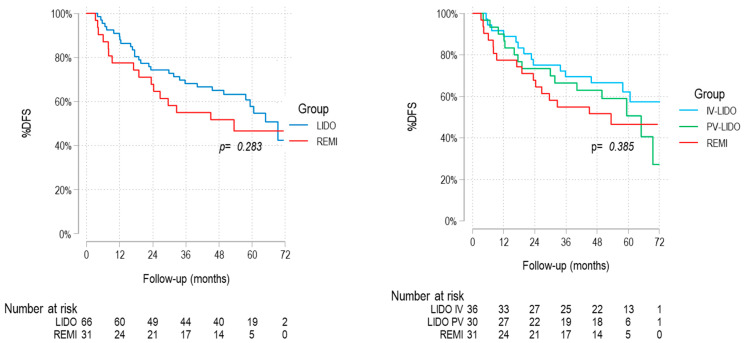
Kaplan–Meier DFS curves. IV, intravenous; PV, paravertebral; LIDO, lidocaine; REMI, remifentanil.

No significant differences in DFS were observed between the lidocaine and remifentanil groups, as indicated by the Kaplan–Meier curves (Figure 3, Table 4). From 54 months onward, an increased cumulative incidence of tumor recurrences was observed in the lidocaine group, mainly attributable to newly diagnosed second primary malignancies (Table 5).

### 3.4. DFS Limited to Recurrences of Malignancies Present at the Time of Surgery

**Figure 4:**
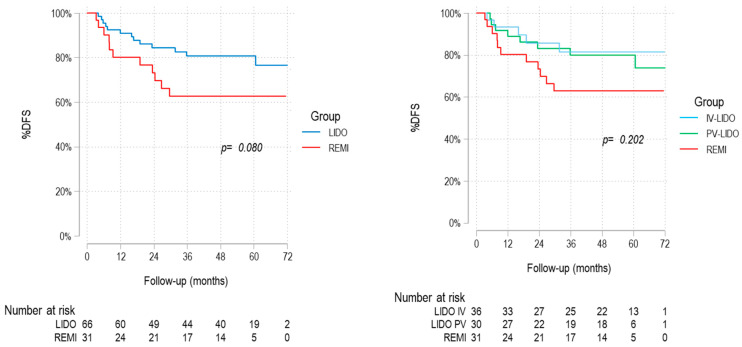
Kaplan–Meier “DFS limited to recurrences of malignancies present at the time of surgery” curves.

A non-significant trend toward improved DFS in lidocaine patients was noted when focusing on recurrences of cancers present at the time of surgery (including the resected lung cancer or prior malignancies) (log-rank *p* = 0.080) (Figure 4). No significant differences between the lidocaine and remifentanil groups were found after adjusting for potential confounders in the multivariate Cox regression analysis (Table 6).

## 4. Discussion

In this post hoc analysis, lidocaine administration during oncologic lung resection was associated with improved OS in primary lung cancer patients, with a more than 60% reduction in the risk of death. No differences in DFS were observed between groups; however, a non-significant trend toward improved DFS in lidocaine patients was noted when focusing on recurrences of cancers present at the time of surgery.

The groups in our analysis showed a homogeneous distribution of comorbidities, surgical characteristics, and oncologic characteristics, as ensured by random allocation within the context of an RCT. The only observed differences were related to postoperative complications. Patients in the lidocaine group experienced fewer pulmonary and cardiac complications, as well as a reduced incidence of major complications. In our RCT, intraoperative administration of lidocaine—whether IV or PV—was associated with both a quantitative and qualitative reduction in postoperative complications, as assessed by the Clavien–Dindo classification. This effect may be attributed to the attenuation of systemic and pulmonary inflammation, as evidenced by serial blood and bronchoalveolar lavage (BAL) samples [13].

Postoperative pulmonary complications have been associated with prolonged postoperative recovery, increased Intensive Care Unit (ICU) admissions, and significantly higher mortality rates [18]. In a previous study from our group, the occurrence of postoperative pulmonary complications was also associated with increased one-year mortality [19].

In the context of oncologic surgery, an optimal immediate postoperative course may have a particularly significant impact on long-term outcomes. Prompt recovery of a competent immune system may help shorten the window of vulnerability during which residual cancer cells could seed and proliferate. Moreover, delayed initiation of adjuvant chemotherapy following surgery has been associated with poorer survival outcomes [20]. Therefore, earlier recovery facilitated by ERAS programs may improve long-term cancer prognosis; however, current evidence remains insufficient [21]. Specifically, in lung cancer, implementation of ERAS pathways has been linked to a shorter interval to the intended oncologic therapy (RIOT) and higher completion rates of at least four chemotherapy cycles [22].

Few studies have assessed the impact of LAs on cancer outcomes specifically in the context of LRS, and none have demonstrated clear benefits. Evaluated LA and route of administration combinations include two prospective randomized trials using ropivacaine via PV [23] and epidural routes [9] as well as two retrospective studies with bupivacaine or ropivacaine administered epidurally [24,25].

A recent clinical study reported improved OS following peritumoral lidocaine infiltration before breast cancer surgery [26]. Despite methodological limitations, such as the absence of a placebo group, this study highlights the importance of attaining an early and effective LA concentration at the tumor site.

The immunomodulatory effects of LAs have been linked to plasma concentrations typically achieved through IV or epidural administration [27]. Notably, lidocaine is the only LA approved for IV administration. In our RCT, both IV and PV routes resulted in a similar ratio between the administered lidocaine dose (adjusted for patient weight and surgical duration) and the serum lidocaine concentration measured at the end of surgery [13]. These findings support the pooled analysis of all patients receiving lidocaine, regardless of the administration route, and are consistent with the parallel behavior observed in the Kaplan–Meier survival curves between the lidocaine groups.

In vitro studies show that LAs reduce survival and proliferation in various cancer cell lines, including lung cancer cells [7]. Although voltage-gated sodium channels (VGSCs) are functionally expressed in many cancer types, including NSCLC [28], most proposed antitumoral mechanisms of LAs appear independent of the VGSC blockade. For instance, lidocaine inhibited lung cancer cell migration induced by tumor necrosis factor alpha (TNF-α) by blocking Src (Src family of protein tyrosine kinases) signaling, a key pathway in inflammatory vascular hyperpermeability [29]. Moreover, LA injection into tumors implanted in immunocompetent mice decreased tumor growth and increased survival, an effect not seen in immunodeficient knock-out mice, suggesting that immunomodulation may significantly contribute to the antitumor effects of LAs and their potential synergistic role with other oncological therapies [30].

No statistically significant differences were observed between groups in overall DFS, which included relapse of the current lung cancer, recurrence of a previous malignancy, or the development of any new primary cancer. While this broad definition increases sensitivity, it limits clinical and prognostic specificity. Recurrence is common in lung cancer due to genetic susceptibility, tobacco use, environmental exposures, and the effects of oncologic treatments themselves. Distinguishing a second primary lung cancer from intrapulmonary metastasis relies on histologic subtype, anatomic location, disease-free interval [31], and, increasingly, molecular profiling [32]. In our subanalysis of cancers present at the time of surgery, we observed a non-significant trend toward improved DFS in patients receiving lidocaine, suggesting a possible protective effect during this critical period.

One limitation of this analysis is its post hoc design, as the original sample size was not powered for long-term outcomes, potentially affecting result consistency. While multivariate models and subgroup analyses help control confounding, they generally require large event numbers for reliability. Nonetheless, the randomized controlled trial setting ensured a reasonably balanced distribution of known confounders across groups.

The heterogeneity of lung cancer types and stages within the study population is another limitation, as these factors are associated with varying prognoses and follow-up protocols. Outcome assessment remained objective, as it was based on clinical and imaging data from electronic medical records, with investigators blinded to group assignment. However, due to the low representation of some subtypes, specific effects cannot be ruled out.

## 5. Conclusions

In this post hoc analysis, lidocaine administration during oncologic lung resection was associated with improved OS in primary lung cancer patients. No differences in DFS were observed between groups; however, a non-significant trend toward improved DFS in lidocaine patients was noted when focusing on recurrences of cancers present at the time of surgery. Further investigation in larger prospective studies is warranted.

## Figures and Tables

**Table 1:** Patient characteristics.

	IV-LIDO (*n* = 36)	PV-LIDO (*n* = 30)	Lidocaine (IV + PV) (*n* = 66)	Remifentanil (*n* = 31)
Demographics				
Sex (Female)	16 (44.4%)	12 (40.0%)	28 (42.4%)	16 (51.6%)
Age (years)	67 (60–77)	65 (59–75)	67 (60–75)	69 (61–75)
Age > 80 years (vs. ≤80)	5 (13.9%)	5 (16.7%)	10 (15.2%)	0 (0.0%)
BMI (kg/m^2^)	27.6 (24.0–31.6)	26.9 (24.4–30.7)	27.5 (24.2–31.3)	28.1 (22.1–32.0)
Comorbidities				
ASA classification:				
ASA I	1 (2.8%)	0 (0.0%)	1 (1.5%)	1 (3.2%)
ASA II	17 (47.2%)	8 (26.7%)	25 (37.9%)	13 (41.9%)
ASA III	18 (50.0%)	21 (70.0%)	39 (59.1%)	16 (51.6%)
ASA IV	0 (0.0%)	1 (3.3%)	1 (1.5%)	1 (3.2%)
ASA > II (vs. I–II)	18 (50.0%)	22 (73.3%)	40 (60.6%)	17 (54.8%)
ARISCAT high risk score (vs. intermediate)	30 (83.3%)	24 (80.0%)	54 (81.8%)	27 (87.1%)
Smoker or ex-smoker (vs. non-smoker)	27 (75.0%)	27 (90.0%)	54 (81.8%)	24 (77.4%)
Alcohol abuse	1 (2.8%)	0 (0.0%)	1 (1.5%)	1 (3.2%)
Hypertension	17 (47.2%)	19 (63.3%)	36 (54.5%)	18 (58.1%)
Diabetes	8 (22.2%)	3 (10.0%)	11 (16.7%)	11 (35.5%)
Dyslipidemia	12 (33.3%)	14 (46.7%)	26 (39.4%)	15 (48.4%)
Surgery characteristics				
Lobectomy (vs. segmentectomy)	27 (75.0%)	22 (73.3%)	49 (74.2%)	28 (90.3%)
Surgery duration (min)	240 (200–275)	240 (180–290)	240 (195–285)	240 (200–290)
Surgery > 180 min (vs. ≤180 min)	29 (80.6%)	22 (73.3%)	51 (77.3%)	25 (80.6%)
Thoracotomy conversion	3 (8.3%)	3 (10.0%)	6 (9.1%)	3 (9.7%)
Intraoperative blood product transfusion	0 (0.0%)	0 (0.0%)	0 (0.0%)	0 (0.0%)
1st month postoperative course				
Any complication	16 (44.4%)	15 (50.0%)	31 (47.0%)	20 (64.5%)
Major complication (CD classification)	2 (5.6%)	1 (3.3%)	3 (4.5%)	6 (19.4%) *
Pulmonary complication	9 (25.0%)	9 (30.0%)	18 (27.3%)	16 (51.6%) *
Cardiac complication	1 (2.8%)	2 (6.7%)	3 (4.5%)	2 (6.5%) *
Postoperative blood product transfusion	2 (5.6%)	1 (3.3%)	3 (4.5%)	0 (0.0%)
1st month mortality	0 (0.0%)	0 (0.0%)	0 (0.0%)	0 (0.0%)
Oncologic characteristics				
Adjuvant treatments:				
Preoperative CT	0	0	0	1 (3.2%)
Preoperative RT	0	0	0	0
Preoperative IT	0	0	0	1 (3.2%)
Postoperative CT	7 (19.4%)	3 (10.0%)	10 (15.2%)	8 (25.8%)
Postoperative RT	3 (8.3%)	0 (0.0%)	3 (4.5%)	2 (6.5%)
Postoperative IT	0	0	0	1 (3.2%)
Type of lung cancer:				
Adenocarcinoma	21 (58.3%)	24 (80.0%)	45 (68.2%)	19 (61.3%)
Squamous cell carcinoma	8 (22.2%)	2 (6.7%)	10 (15.2%)	7 (22.6%)
Adenosquamous	3 (8.3%)	1 (3.3%)	4 (6.1%)	1 (3.2%)
Neuroendocrine	3 (8.3%)	3 (10.0%)	6 (9.1%)	2 (6.5%)
NOS/Anaplastic	0 (0.0%)	0 (0.0%)	0 (0.0%)	2 (6.5%)
Small-cell carcinoma	1 (2.8%)	0 (0.0%)	1 (1.5%)	0 (0.0%)
Lung cancer stage:				
IA	17 (47.2%)	22 (73.3%)	39 (59.1%)	15 (48.4%)
IB	10 (27.8%)	5 (16.7%)	15 (22.7%)	5 (16.1%)
IIA	1 (2.8%)	0 (0.0%)	1 (1.5%)	4 (12.9%)
IIB	2 (5.6%)	2 (6.7%)	4 (6.1%)	2 (6.5%)
IIIA	5 (13.9%)	1 (3.3%)	6 (9.1%)	5 (16.1%)
IIIB	1 (2.8%)	0 (0.0%)	1 (1.5%)	0 (0.0%)
Lung cancer stage > I (vs. I)	9 (25.0%)	3 (10.0%)	12 (18.2.8%)	11 (35.5%)
Stage T:				
T in situ/microinvasive	2 (5.6%)	3 (10.0%)	5 (7.6%)	0 (0.0%)
T1	17 (47.2%)	19 (63.3%)	36 (54.5%)	17 (54.8%)
T2	15 (41.7%)	5 (16.7%)	20 (30.3%)	11 (35.5%)
T3	2 (5.6%)	3 (10.0%)	5 (7.6%)	2 (6.5%)
T4	0 (0.0%)	0 (0.0%)	0 (0.0%)	1 (3.2%)
T > 1 (vs. T1)	17 (47.2%)	8 (26.7%)	25 (37.9%)	14 (45.2%)
Stage N:				
N0	29 (80.6%)	29 (96.7%)	58 (87.9%)	27 (87.1%)
N1	1 (2.8%)	1 (3.3%)	2 (3.0%)	0 (0.0%)
N2	6 (16.7%)	0 (0.0%)	6 (9.1%)	4 (12.9%)
N3	0 (0.0%)	0 (0.0%)	0 (0.0%)	0 (0.0%)
N > 0 (vs. N0)	7 (19.4%)	1 (3.3%)	8 (12.1%)	4 (12.9%)

Data are expressed as median (interquartile range) or number (%). Some variables were dichotomized for Cox analysis; ‘vs.’ indicates the reference group. IV, intravenous; PV, paravertebral; LIDO, lidocaine; REMI, remifentanil; ASA, American Society of Anesthesiologists Physical Status Classification System; BMI, body mass index; CT, chemotherapy; RT, radiotherapy; IT, immunotherapy; NOS, not otherwise specified; ARISCAT, Assess Respiratory Risk in Surgical Patients in Catalonia [16]; CD, Clavien–Dindo classification [15]. The lung cancer stage refers to the Eighth Edition Lung Cancer Stage Classification, in effect at the time of our analysis [17]. * *p* < 0.05 LIDO vs. REMI.

**Table 2:** Multivariate and Univariate Cox Regression Analysis for OS.

Variable	Univariate		Multivariate A		Multivariate B	
	HR (95% CI)	*p*-Value	HR (95% CI)	*p*-Value	HR (95% CI)	*p*-Value
LIDO_REMI (Remi vs. Lido)	2.62 (1.12–6.12)	0.026	2.59 (1.13–5.96)	0.025	5.41 (1.86–15.72)	0.002
Age > 80 years (vs. ≤ 80)	3.12 (1.20–8.10)	0.019	-	-	8.30 (2.20–31.27)	0.002
ASA > II (III–IV vs. I–II)	3.36 (1.13–10.04)	0.030	2.75 (0.92–8.19)	0.070	2.70 (0.89–8.23)	0.081
Duration >180 min (vs. ≤180 min)	0.50 (0.20–1.22)	0.128	0.48 (0.20–1.12)	0.091	-	-
Lung cancer stage > I (vs. I)	2.97 (1.25–7.05)	0.014	2.30 (0.96–5.50)	0.061	-	-
Stage N > 0 (vs. 0)	3.15 (1.09–9.08)	0.034	-	-	4.51 (1.52–13.40)	0.007

LIDO, lidocaine; REMI, remifentanil; ASA, American Society of Anesthesiologists Physical Status Classification System; HR, hazard ratio; CI, confidence interval.

**Table 3:** Causes of Death by Time Interval.

Time Interval	Cause of Death	Lidocaine (*n* = 66)	Remifentanil (*n* = 31)
1st-Year Deaths	Cancer progression—lung	2	2
	Cancer progression—other	0	1 (previous prostate)
	Non-cancer cause	0	1 (pneumonia)
	Total events	2	4
Deaths Between 1 and 3 Years	Cancer progression—lung	2	0
	Cancer progression—other	0	1 (previous ovarian)
	Non-cancer cause	1 (pneumonia)	1 (stroke)
	Total events	3	2
Deaths After 3 Years	Cancer progression—lung	2	2
	Cancer progression—other	1 (new bladder)	0
	Non-cancer cause	2 (renal insufficiency, cerebellar hemorrhage)	3 (pneumonia, pancreatitis, Alzheimer’s disease)
	Total events	5	5

**Table 4:** Multivariate and Univariate Cox Regression Analysis for DFS.

Variable	Univariate		Multivariate A		Multivariate B	
	HR (95% CI)	*p*-Value	HR (95% CI)	*p*-Value	HR (95% CI)	*p*-Value
LIDO_REMI (Remi vs. Lido)	1.40 (0.75–2.60)	0.292	1.29 (0.69–2.39)	0.428	1.49 (0.78–2.84)	0.225
ASA > II (III–IV vs. I–II)	1.05 (0.59–1.88)	0.866	0.93 (4.98–1.74)	0.829	-	-
Lobectomy (vs. Segmentectomy)	0.57 (0.29–1.10)	0.096	-	-	0.47 (0.23–0.94)	0.033
Duration > 180 min (vs. ≤180 min)	0.92 (0.47–1.79)	0.796	0.89 (0.43–1.85)	0.756	-	-
Lung cancer stage > I (vs. I)	1.78 (0.95–3.36)	0.074	1.74 (0.90–3.36)	0.101	1.83 (0.96–3.51)	0.069

LIDO, lidocaine; REMI, remifentanil; ASA, American Society of Anesthesiologists Physical Status Classification System; HR, hazard ratio; CI, confidence interval.

**Table 5:** Cancer Recurrence by Time Interval and Origin.

Time Interval	Type of Recurrence	Lidocaine (*n* = 66)	Remifentanil (*n* = 31)
1st-Year Recurrences	Same lung cancer	5	4
	Other previous cancer	0	1
	Second primary lung cancer	2	1
	Second primary non-lung cancer	0	0
	Total events	7	6
Recurrences Between 1 and 3 Years	Same lung cancer	5	3
	Other previous cancer	2	1
	Second primary lung cancer	3	1
	Second primary non-lung cancer	4	1
	Total events	14	6
Recurrences After 3 Years	Same lung cancer	1 (1 after >54 months)	0
	Other previous cancer	0	2
	Second primary lung cancer	5 (3 after >54 months)	0
	Second primary non-lung cancer	2 (2 after >54 months)	1 (1 after >54 months)
	Total events	8	3

**Table 6:** Multivariate and Univariate Cox Regression Analysis for “DFS limited to recurrences of malignancies present at the time of surgery”.

Variable	Univariate		Multivariate A		Multivariate B	
	HR (95% CI)	*p*-Value	HR (95% CI)	*p*-Value	HR (95% CI)	*p*-Value
LIDO_REMI (Remi vs. Lido)	2.02 (0.91–4.48)	0.083	1.67 (0.75–3.73)	0.211	2.10 (0.89–4.97)	0.091
ASA > II (III–IV vs. I–II)	0.77 (0.35–1.69)	0.511	0.52 (0.22–1.24)	0.141		
Lobectomy (vs. Segmentectomy)	0.44 (0.19–1.03)	0.058	-	-	0.26 (0.11–0.65)	0.004
Duration > 180 min (vs. ≤180 min)	0.69 (0.29–1.61)	0.390	0.55 (0.21–1.43)	0.217		
Postoperative transfusion	3.69 (0.85–16.08)	0.082	-	-		
Postoperative CT	2.97 (1.29–6.83)	0.011	-	-	2.47 (0.78–7.88)	0.126
Postoperative RT	2.90 (0.90–9.38)	0.076	-	-		
Lung cancer stage > I (vs. I)	2.80 (1.24–6.32)	0.014	3.08 (1.38–6.87)	0.006	1.76 (0.57–5.36)	0.326
Stage N > 0 (vs. N0)	2.56 (1.04–6.30)	0.040	-	-		

LIDO, lidocaine; REMI, remifentanil; ASA, American Society of Anesthesiologists Physical Status Classification System; CT, chemotherapy; RT, radiotherapy; HR, hazard ratio; CI, confidence interval.

## Data Availability

Data available on request due to restrictions (regarding patient confidentiality).

## Abbreviations

The following abbreviations are used in this manuscript (in alphabetical order):

ARISCAT	Assess Respiratory Risk in Surgical Patients in Catalonia
ASA	American Society of Anesthesiologists Physical Status Classification System
BAL	Bronchoalveolar Lavage Fluid
BMI	Body Mass Index
CD	Clavien Dindo classification
CI	Confidence Interval
CT	Chemotherapy
DFS	Disease-Free Survival
ERAS	Enhance Recovery After Surgery program
HR	Hazard Rate
ICU	Intensive Care Unit
IT	Immunotherapy
IV	Intravenous
LA	Local Anesthetic
LIDO	Lidocaine
LRS	Lung Resection Surgery
NOS	Not Otherwise Specified Lung Cancer
NSAID	Nonsteroidal Anti-Inflammatory Drugs
NSCLC	Non-Small Cell Lung Cancer
OLV	One-Lung Ventilation
OS	Overall Survival
PEEP	Positive End Expiration Pressure
PMN	Polymorphonuclear Lymphocytes
PV	Paravertebral
RCT	Randomized Controlled Trial
REMI	Remifentanil
RIOT	Return to Intended Oncologic Therapy
RT	Radiotherapy
Src	Src Family of Protein Tyrosine Kinases
SSR	Surgical Stress Response
Th	T helper cell type
TLV	Two-Lung Ventilation
TNF	Tumor Necrosis Factor
VATS	Video-Assisted Thoracoscopy
VGSC	Voltage-Gated Sodium Channels
